# Arbutin increases *Caenorhabditis elegans* longevity and stress resistance

**DOI:** 10.7717/peerj.4170

**Published:** 2017-12-20

**Authors:** Lin Zhou, Xueqi Fu, Liyan Jiang, Lu Wang, Shuju Bai, Yan Jiao, Shu Xing, Wannan Li, Junfeng Ma

**Affiliations:** School of Life Sciences, Jilin University, Changchun, Jilin Province, China

**Keywords:** Arbutin, *C. elegans*, *daf-16*, Stress, Longevity

## Abstract

Arbutin (*p*-hydroxyphenyl-β-D-glucopyranoside), a well-known tyrosinase inhibitor, has been widely used as a cosmetic whitening agent. Although its natural role is to scavenge free radicals within cells, it has also exhibited useful activities for the treatment of diuresis, bacterial infections and cancer, as well as anti-inflammatory and anti-tussive activities. Because function of free radical scavenging is also related to antioxidant and the effects of arbutin on longevity and stress resistance in animals have not yet been confirmed, here the effects of arbutin on *Caenorhabditis elegans* were investigated. The results demonstrated that optimal concentrations of arbutin could extend lifespan and enhance resistance to oxidative stress. The underlying molecular mechanism for these effects involves decreased levels of reactive oxygen species (ROS), improvement of *daf-16* nuclear localization, and up-regulated expression of *daf-16* and its downstream targets, including *sod-3* and *hsp16.2*. In this work the roles of arbutin in lifespan and health are studied and the results support that arbutin is an antioxidant for maintaining overall health.

## Introduction

Tannins, also known as plant polyphenols, comprise the most common category of secondary metabolites and are present in all vegetative organs of flowering plants (Scalbert et al., 2005). Studies have shown that plant polyphenols mainly participate in plant chemical defenses by interfering with normal functions of various macromolecules (Spencer et al., 2008) and also exhibit antioxidant, anti-inflammatory, antibacterial, antitumor and other biological activities (Balandrin et al., 1985).

Arbutin (C_12_H_16_O_7_), a plant polyphenol with a simple molecular structure, is widely distributed in animals, plants and microbes. It exhibits an acicular crystal habit and can be processed into a white or grey powder. Arbutin can dissolve in methyl alcohol, ethyl alcohol, acetonitrile and tetrahydrofuran, but it is insoluble in solvents such as cyclohexane, diethyl ether, chloroform, petroleum and DMSO. It is unstable and easily hydrolyzed in an acid environment, but it has been successfully isolated using plant extraction techniques, biological transformation, organic synthesis and enzymatic synthesis methods (Seo et al., 2012). Arbutin possesses two functional groups, a hydrophilic anhydroglucose group and a melanin synthase inhibitory phenolic group. The latter group inhibits melanin synthase to lighten hair and it is the reason that arbutin was widely used in the cosmetic hairdressing industry. Furthermore, recent reports have showed that arbutin exhibited many biological activities, including antioxidant, diuretic, antibacterial, anti-inflammatory, anti-tussive, anticancer and so on (Mustapha et al., 2016).

As an animal model for human biomedical research, *Caenorhabditis elegans* presents many advantages, such as easy culture and rapid reproduction with short generation times. Moreover, the organism is visible, due to its translucent body that allows fluorescence labeled organs to be easily visualized. Furthermore, this organism exist numerous recombinant strains which incorporate GFP reporter genes for many important cellular pathways, including aging, oxidative stress tolerance and many diseases (Abbas & Wink, 2010; Henderson & Johnson, 2001; Hsu, Murphy & Kenyon, 2003; Link et al., 2015). Finally, *C. elegans* is a suitable tool for the study of human health conditions and diseases because its homologues have been identified for 60–80% of human genes (Kaletta & Hengartner, 2006).

In this study, we investigated the effects of arbutin on *C. elegans* longevity and stress resistance and evaluated the signaling pathways involved.

## Materials and Methods

### Chemicals and reagents

Juglone, DCFH-DA and sodium azide were purchased from TransGen Biotech Co., Ltd. (Beijing, China). All other chemicals were of analytical or reagent grade.

### *C. elegans* strains and maintenance

The following strains in this study were obtained from the Caenorhabditis Genetics Center: wild type N2, CF1038 (daf-16 (mu86)I), TJ356 zIs356 (daf-16p::daf-16a/b::GFP+rol-6(su1006)). Worms were maintained at 20 °C on agar plates containing nematode growth medium (NGM, 1.7% agar, 25 mM potassium phosphate, pH 6.0, 50 mM NaCl, 2.5 µg/ml peptone, 5 µg/ml cholesterol, 1 mM MgSO_4_, 1 mM CaCl_2_) seeded with *Escherichia coli* OP50. Arbutin (Wuhan Fude Chemical Co., Ltd., Wuhan City, Hubei Province, China; >98% pure, HPLC grade) dissolved in ultrapure water to a concentration of 50 mM and added directly to *E. coli* OP50 cultured in LB liquid medium supplement present to final concentrations of 0.5, 2.5, 5, 10 and 20 mM.

### Lifespan assay

The lifespan assay of *C. elegans* was investigated as previously described (Schlotterer et al., 2009). The pre-fertile period of adulthood was used as time zero (*t* = 0). The nematodes were maintained on NGM plates containing either various concentrations of arbutin or vehicle (control) from birth and transferred to new plates every day. Worms were recorded as dead if they did not move after repeated stimulus and they were excluded if they crawled away from the plate. The assays were running until all animals died. Experiments were performed in at least triplicate with 120 nematodes each at 20 °C. All subsequent assays were conducted using the most effective concentration in inducing lifespan extension relative to wild type controls.

### Stress resistance assays

Twenty gravid adult nematodes (N2 or CF1038) were placed on NGM plates seeded with *E. coli* strain OP50 together with no arbutin (control group) or 5 mM arbutin (experimental group). Worms were allowed to lay eggs at 20 °C for approximately 2 h to obtain a synchronous population. Then, they were removed and the plates were placed back at 20 °C until the progeny reached young adulthood (about 72 h). Worms were transferred to fresh control plates or arbutin plates every day. On the fifth day (at about 120 h), animals were submitted to various kinds of stressors. Experiments were performed in triplicate, with 60 nematodes each at least.

In the heat shock stress assay, animals were transferred from 20 °C to 35 °C incubator and the number of surviving worms were recorded every hour until all worms died. In the juglone stress assay, worms were placed in 96-well plates containing 200 µl S medium (100 mM NaCl, 0.01 mM cholesterol and 50 mM potassium phosphate, pH 6.0) with 200 µM juglone in each well (Waterston & Brenner, 1978). Worms were observed at 20 °C every hour until no worms remained alive. In the UV-irradiation stress assay, worms were treated with 254 nm UVC irradiation with a dose of 1,000 J/m^2^ for 8 min 40 s and immediately transferred to 20 °C incubator. Animals were counted every 12 h until they died.

### Measurement of general ROS levels

Five-day-old worms with or without 5 mM arbutin treatment from birth were transferred into 100 µl M9 buffer (22 mM KH_2_PO_4_, 42 mM Na_2_HPO_4_, 85 mM NaCl, 1 mM MgSO_4_) with 200 µM DCFH-DA. After incubation with DCFH-DA for 2 h, animals were washed with M9 buffer to remove residual DCFH-DA. And then they were transferred to 96-well plates containing 200 µl M9 buffer per well. ROS-associated fluorescence levels were measured using a fluorescence microplate reader (Infinite F200 PRO, Tecan, Switzerland) at 485 nm excitation and 535 nm emission settings. Arbutin-treated group relative fluorescence unit (RFU) was compared with control group of which mean value RFU was set as 1 to reflect relative ROS levels. Ten worms were added to each well and at least twenty parallel wells were performed for each group (control or 5 mM arbutin treatment).

### Fluorescence quantification of DAF-16::GFP

Transgenic strains TJ356 worms harboring the DAF-16::GFP reporter gene were subjected to arbutin treatments from birth to 5 days of age. Then, they were treated with 35 °C heat shock for 30 min. Later, animals were narcotized with 10 µM sodium azide and fixed to glass slides containing 5% agar for observation under a fluorescence microscope. DAF-16 expression corresponded with green fluorescence intensity. Experiments were performed in at least triplicate, with 10 nematodes each.

### Quantitative real-time PCR

After birth followed by 5 days of incubation either with or without 5 mM arbutin, worms were collected and washed with M9 buffer to remove residual *E. coli* OP50 on their skin. Trizol reagent (Thermo Fisher Scientific, Waltham, MA, USA) was used for total RNA extraction. The primers used in RT-PCR were as follows: *daf-16*, 5′-TTTCCGTCCCCGAACTCAA-3′ and 5′-ATTCGCCAACCCATGATGG-3′; *sod-3*, 5′-AGCATCATGCCACCTACGTGA-3′ and 5′-CACCACCATTGAATTTCAGCG-3′; *hsp-16.2*, 5′- CTGCAGAATCTCTCCATCTGAGTC -3′ and 5′-AGATTCGAAGCAACTGCACC -3′; *ama-1*, 5′-CTGACCCAAAGAACACGGTGA-3′ and 5′-TCCAATTCGATCCGAAGAAGC-3′. The gene *ama-1* was used as an internal reference gene and the AB 7500 RT-PCR (Applied Biosystems, Foster City, CA, USA) detection system was used to visualize RT-PCR products. RT-PCR data were analyzed using the comparative 2^−△△Ct^ method (Livak & Schmittgen, 2001). Experiments were performed in at least triplicate, with 200 nematodes per group for total RNA extraction.

### Brood size

Animals synchronized at the first larval stage were grown on NGM/OP50 plates with or without 5 mM arbutin. On the second day, animals were separated to relevant plates with one animal per plate and then they were transferred every 24 h to fresh control or 5 mM arbutin plates until cessation of egg production. The total number of progeny from each animal was counted and the number of progeny for each group was averaged (Li et al., 2008). Experiments were performed in triplicate, with 10 nematodes each.

### Statistical analysis

The data from lifespan of control and arbutin-treated worms (N2 and CF1038) under normal and stress conditions were determined using the Kaplan–Meier survival assay in Prism 5 software (GraphPad Software, Inc., La Jolla, CA, USA) and statistical significance was analyzed by log-rank (Mantel-cox) test. Data other than lifespan were statistically analyzed using one-way analysis of variance (ANOVA). Results were expressed as the mean ± standard error of the mean (SEM). *P*-values <0.05 were taken as statistically significant (0.01 ≤ * *p* < 0.05, 0.001 ≤ ** *p* < 0.01, *** *p* < 0.001).

## Results

### Arbutin extends lifespan of *C. elegans* under normal culture conditions

**Figure 1:**
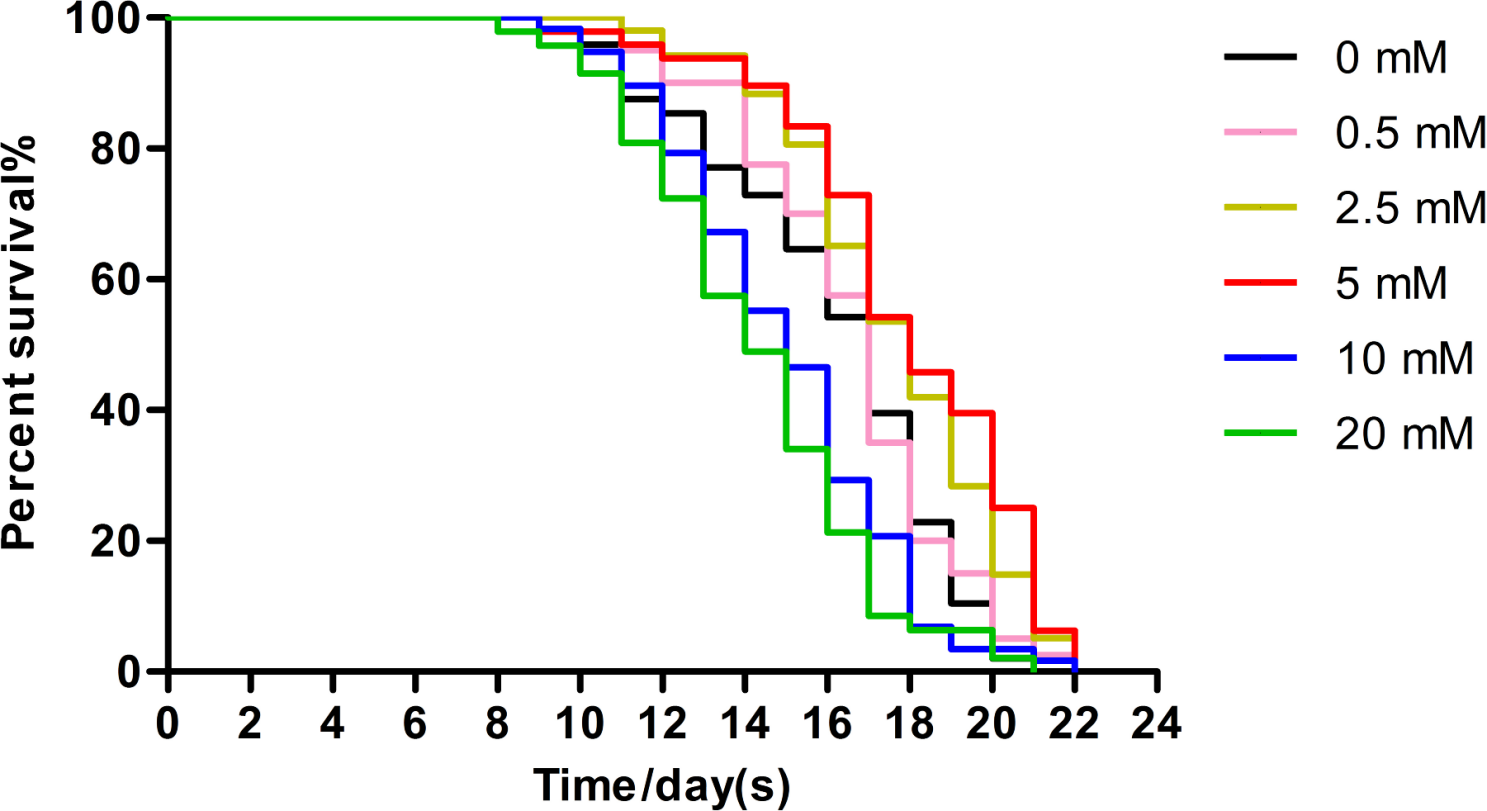
Effect of arbutin on the lifespan in *C. elegans*. Wild type animals (*N* = 120–182 in each group) were treated without (0 mM) or with low (0.5, 2.5 mM), moderate (5 mM) and high (10, 20 mM) concentrations of arbutin at 20 °C from birth, when survival was monitored. The experiment was repeated multiple times and a representative trial was shown. Statistical difference between the curves was analyzed by log-rank test (Fig. 1, *p* < 0.0001).

Low concentrations of arbutin showed dose-related lifespan-extending effects, with the maximum effect observed at 5 mM (extending worm average lifespan by 11.89%), whereas 10 mM and 20 mM arbutin exhibited toxicity. It is certain that arbutin significantly extended lifespan of nematodes in a suitable concentration. We performed additional experiments using 5 mM arbutin to further test its protective effects on worms under various types of stressors (Fig. 1, *p* < 0.0001).

### Arbutin improves worms’ survival under stress

#### Heat stress

**Figure 2:**
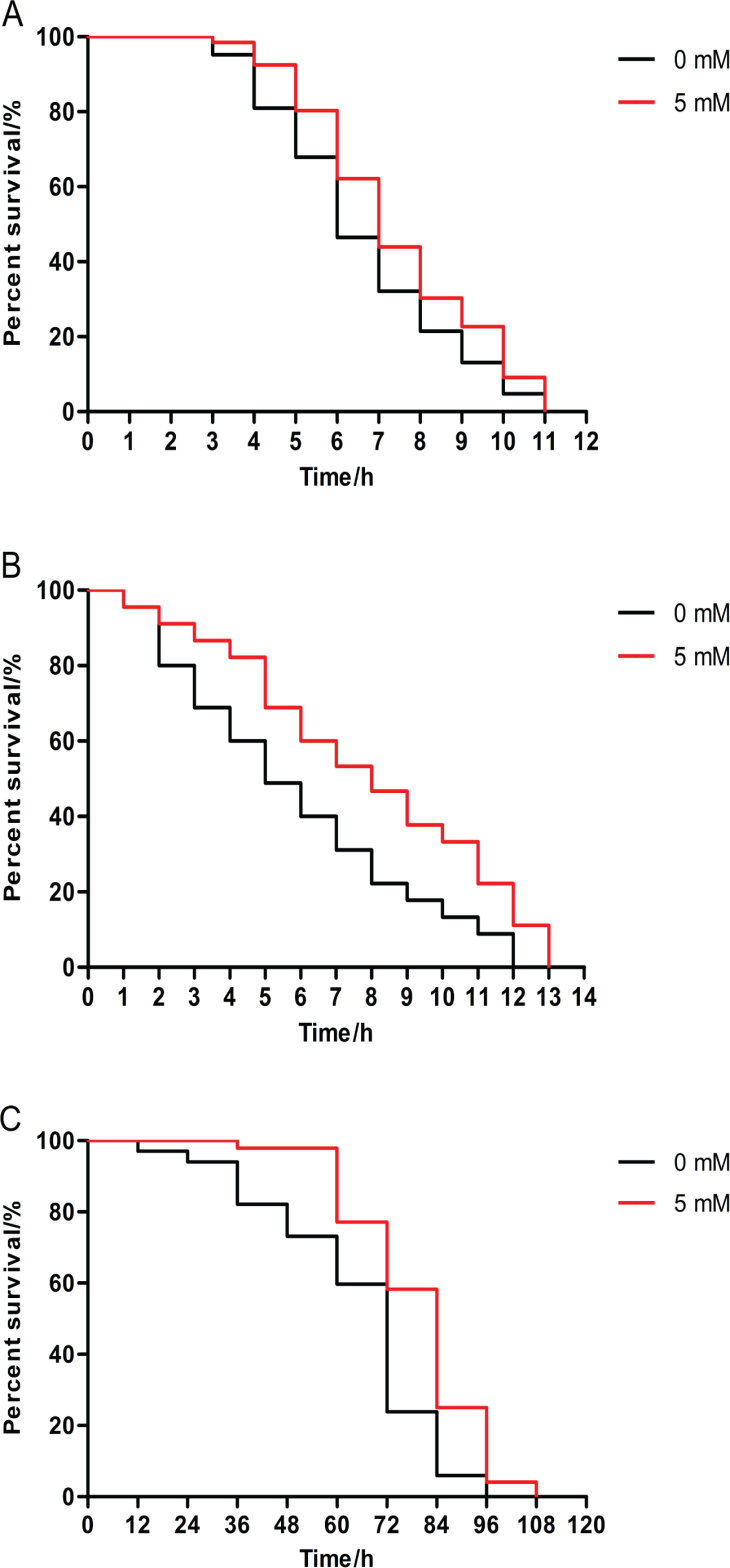
Effect of pretreatment with arbutin on resistance to stress in *C. elegans*. Animals were treated with 5 mM arbutin from birth to 120 h at 20 °C and exposed to a variety of stressors. Worms pretreated with arbutin survived significantly longer than control after (A) 35 °C heat shock (*N* = 66–84 animals, *p* < 0.05), (B) exposure to 200 µM juglone (*N* = 90 animals, *p* < 0.0001) or (C) UV irradiation at 1,000 J/m^2^ (*N* = 68–96 animals, *p* < 0.0001). Each experiment was representative of three independent trials. Statistical difference between the curves was analyzed by log-rank test.

The survival rate of animals pretreated with 5 mM arbutin from birth to 120 h of age had significantly increased relative to the control under 35 °C stress. Results demonstrated that arbutin could enhance resistance to heat shock in *C. elegans* (Fig. 2A, *p* < 0.05).

#### Juglone stress

Additionally, 5 mM arbutin pretreatment also enhanced survival rate compared to untreated worms under oxidative stress conditions induced by juglone from the second day of adulthood (Fig. 2B, *p* < 0.0001).

#### UV stress

Next, we observed 5 mM arbutin-pretreated worms were more irradiation tolerant than untreated control worms, as 5 mM arbutin pretreatment significantly improved animals’ resistance to UV-irradiation relative to the control (Fig. 2C, *p* < 0.0001).

Apparently, protective effects of a low but suitable arbutin concentration were observed under both normal and stress culture conditions, suggesting that suitable concentrations arbutin could promote statistically significant longevity in worms.

### Arbutin reduces the ROS levels

**Figure 3:**
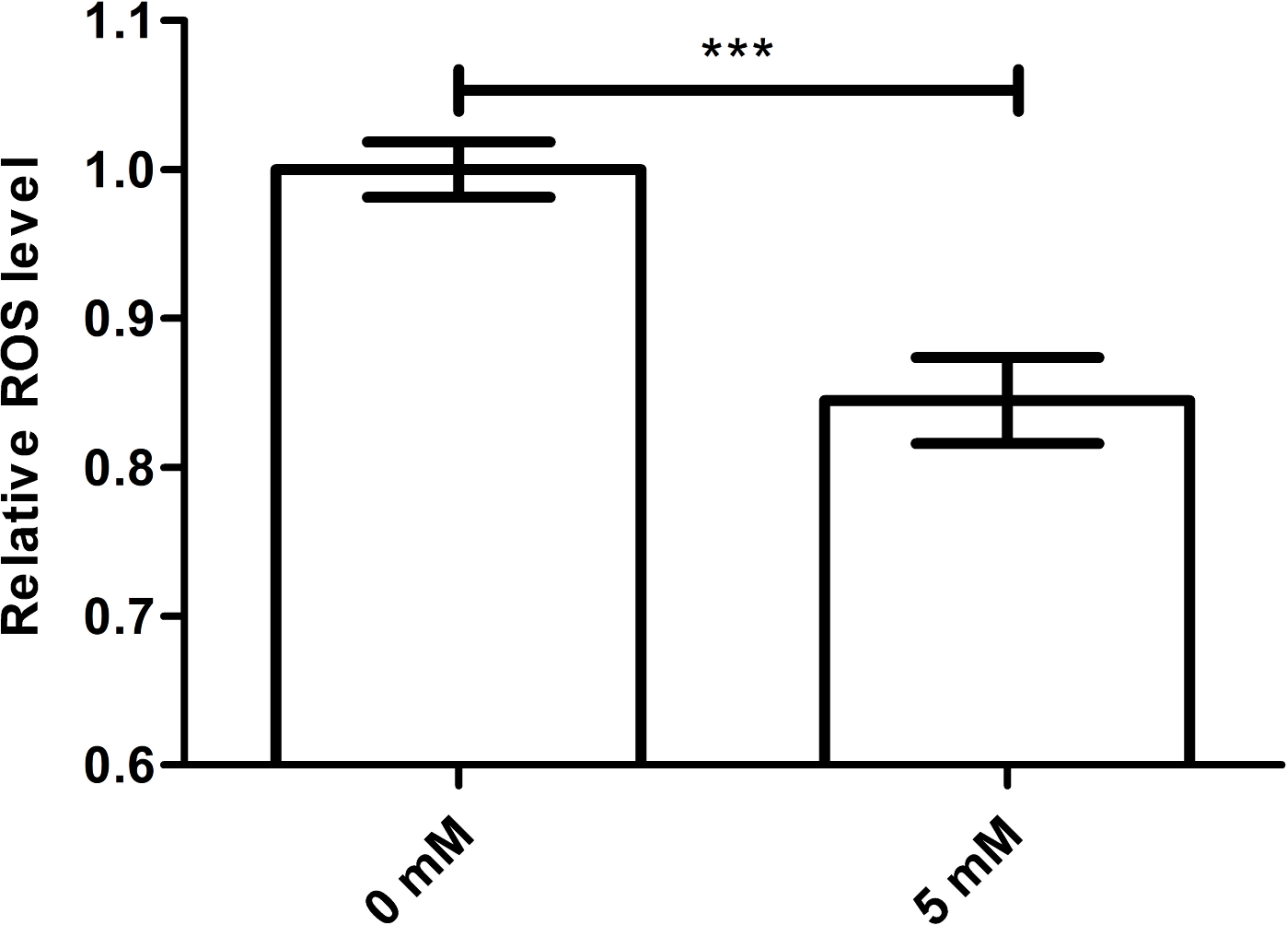
Effect of arbutin on ROS accumulation in *C. elegans*. Wild type N2 animals treated with 5 mM arbutin accumulated less ROS than wild type N2 control animals. *Error bars* represented the standard error of the mean (SEM) (*N* = 24–25 parallel experiments, *p* < 0.001).

It is known that excessive free radical accumulation may harm organisms. Because oxygen, an electron receptor, may promote the formation and accumulation of ROS and cellular respiration is one of the chief causes of oxidative damage in aerobic organisms. Meanwhile, newly generated free radicals, such as superoxide anions, hydrogen peroxide and hydroxyl radicals, are immediately eliminated by antioxidant enzyme systems (Harman, 1956). Therefore, we studied whether the longevity effect of arbutin on worms was associated with ROS scavenging ability. While ROS level was usually higher during a stress response, it was significantly decreased in worms treated with arbutin compared with control. We therefore suggested that arbutin might act as an antioxidant by scavenging free radicals, ultimately extending *C. elegans* lifespan and improving resistance to environment stress (Fig. 3, *p* < 0.001).

### Arbutin has no effect on the resistance to stress of *daf-16* mutant worms

**Figure 4:**
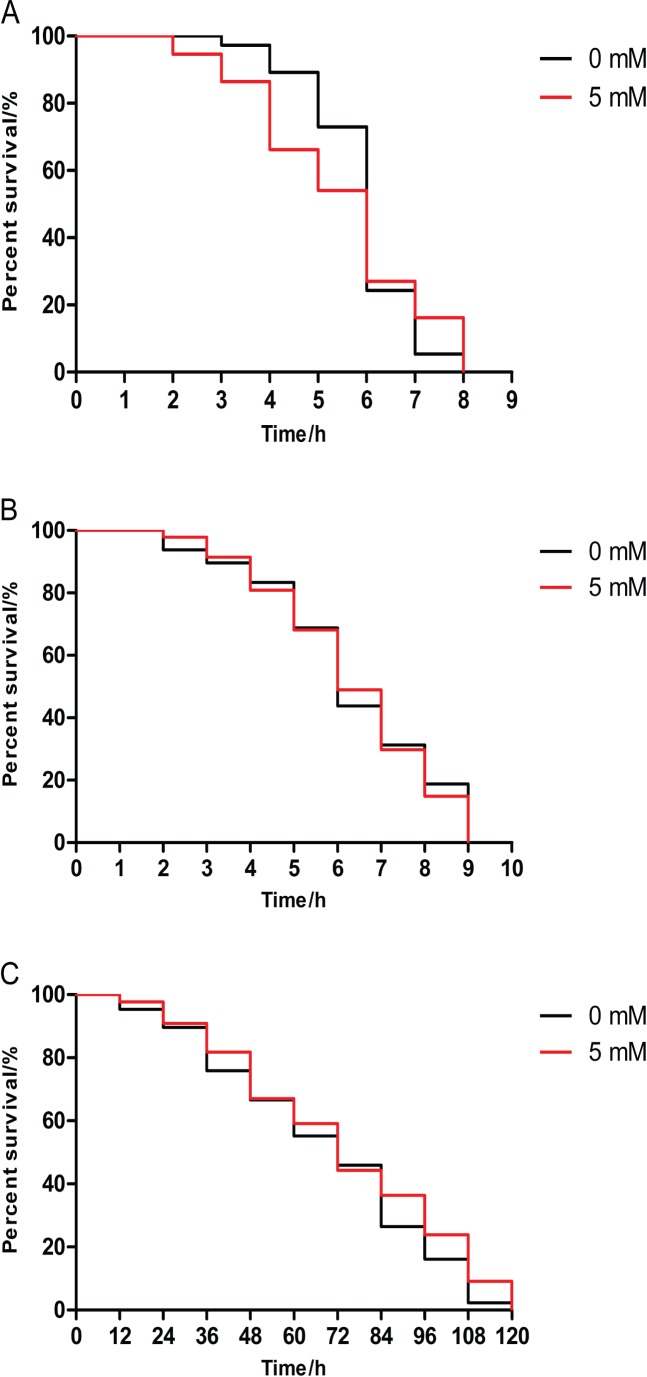
Effect of arbutin on resistance to stress in transgenic strain CF1038 worms. Animals were treated with 5 mM arbutin from birth to 120 h at 20 °C and exposed to a variety of stressors. There was no survival significance between worms pretreated with and without arbutin after (A) 35 °C heat shock (*N* = 74 animals, *p* = 0.6719), (B) exposure to 200 µM juglone (*N* = 96 animals, *p* = 0.7941) or (C) UV irradiation at 1,000 J/m^2^ (*N* = 85–88 animals, *p* = 0.1605). Each experiment was representative of three independent trials. Statistical difference between the curves was analyzed by log-rank test.

DAF-16 is a major transcription factor which modulates longevity and stress resistance in *C. elegans* (Murphy et al., 2003). We examined the lifespan of *daf-16* mutants CF1038 under stress. We found that there was no difference between lifespan of 5 mM arbutin treated and untreated mutant CF1038 under three kinds of stressors (heat shock, juglone and UV-irradiation), indicating that arbutin acting on animals was related to *daf-16* (Figs. 4A–4C, *p* > 0.05).

### Arbutin improves DAF-16::GFP nuclear localization in TJ356 worms

**Figure 5:**
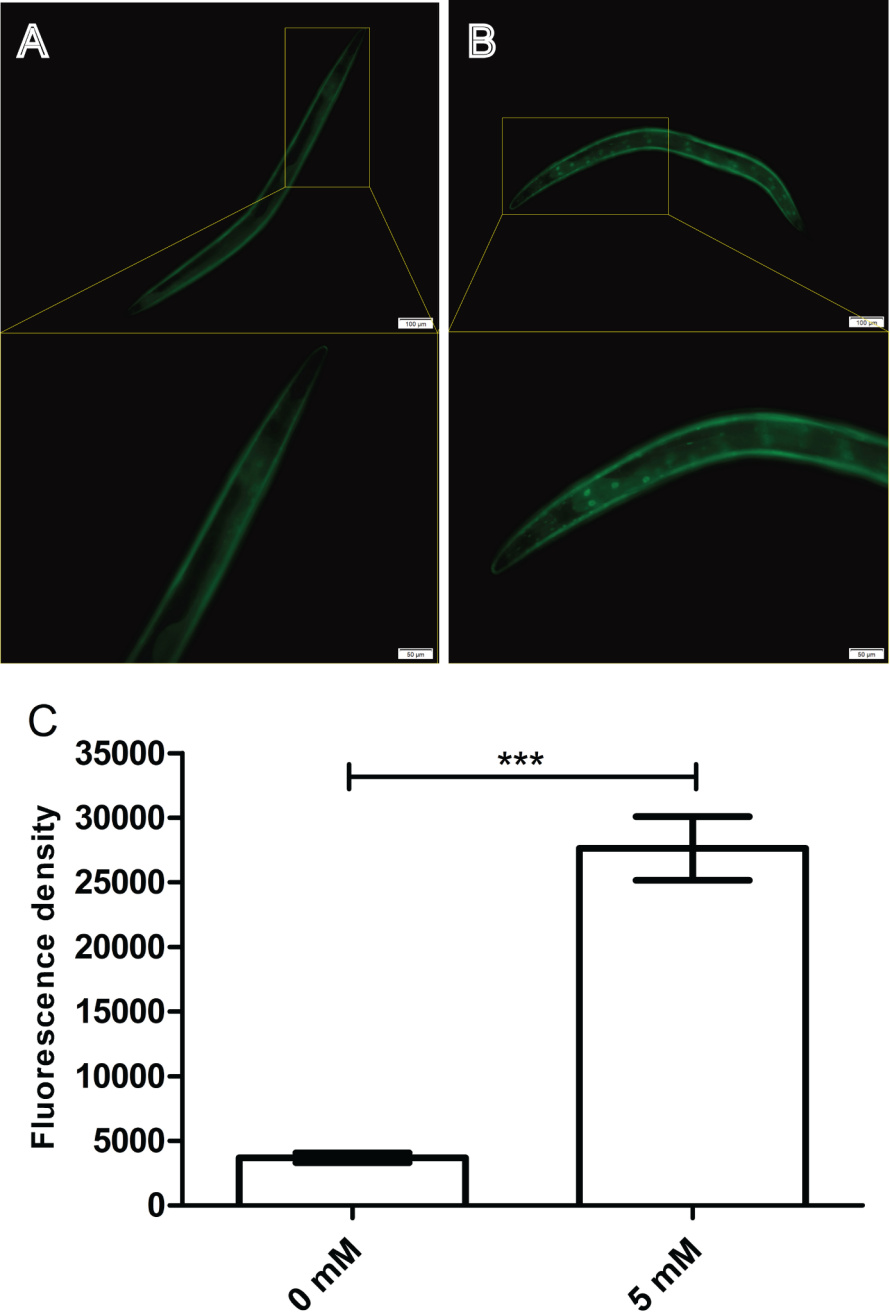
Effect of arbutin on DAF-16::GFP nuclear localization in transgenic strain TJ356. Worms treated with 5 mM arbutin (B) displayed fluorescence intensity which were significantly up-regulated (C) relative to the control group (A). *Error bars* represented the standard error of the mean (SEM) (*N* = 10 animals per group, *p* < 0.001).

The nuclear localization of DAF-16 is essential for its transcriptional activity. Thus, we conjectured that arbutin could also positively regulate DAF-16 expression in nuclear. Here, we employed a transgenic strain TJ356 which expressed DAF-16::GFP (Lee, Hench & Ruvkun, 2001) reporter to quantitatively visualize DAF-16 expression through a fluorescence microscope. After 35 °C heat shock for 30 min, 5 mM arbutin-treated worms displayed a significantly increased fluorescence intensity compared with the control group. These results suggested that arbutin might also activate antioxidant-related signaling pathways that ultimately led to improve DAF-16 nuclear localization for increasing resistance to oxidative stress (Figs. 5A–5C, *p* < 0.001).

### Arbutin promotes relative mRNA levels of *daf-16* and it downstream genes *sod-3* and *hsp-16.2*

**Figure 6:**
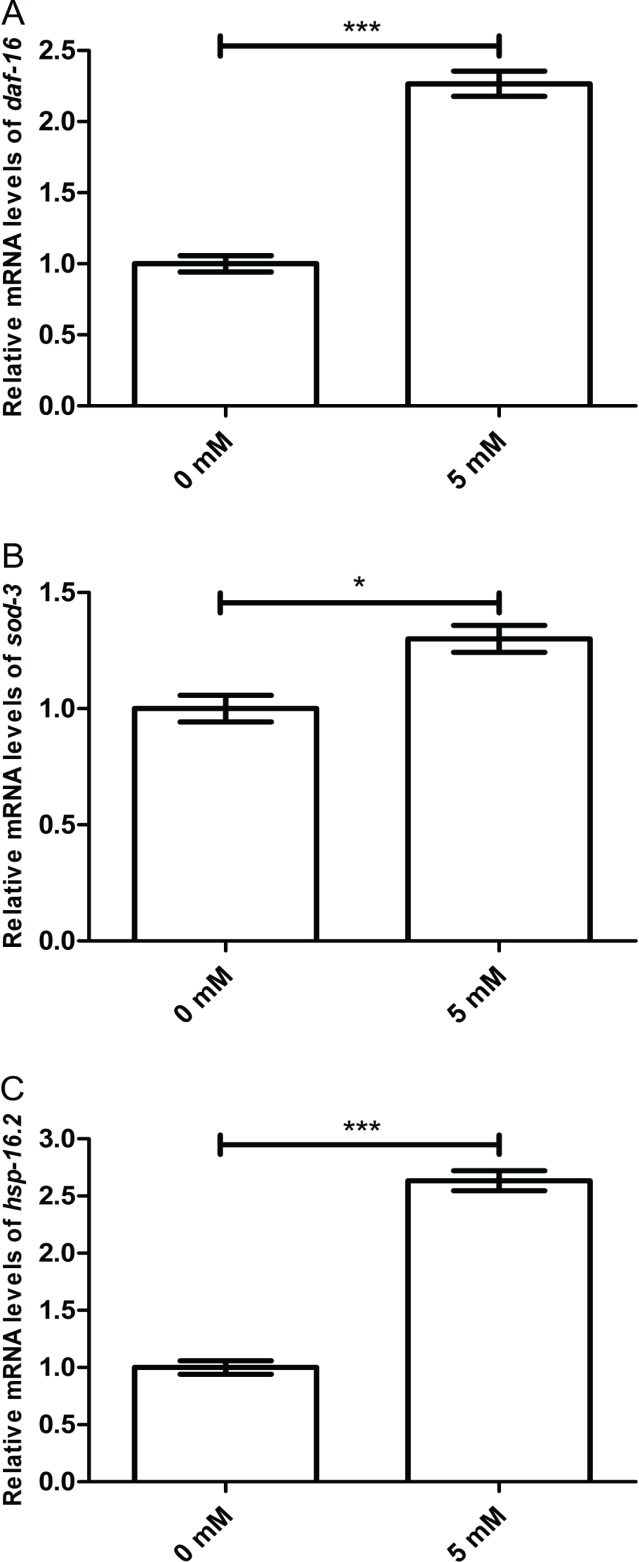
Effect of arbutin on relative mRNA levels of *daf-16*, *sod-3* and *hsp-16.2* in *C. elegans*. (A) *daf-16* (*p* < 0.001) and its downstream targets (B) *sod-3* (*p* < 0.05) and (C) *hsp-16.2* (*p* < 0.001) were all up-regulated with arbutin treatment relative to the control. *Error bars* represented the standard error of the mean (SEM) (*N* = 3 parallel experiments).

To further investigate whether working mechanism of arbutin was regulated by DAF-16 and its target proteins, we measured mRNA levels of *daf-16*, *sod-3* and *hsp-16.2* in N2 worms treated with or without 5 mM arbutin. It is known that protein activities which are dependent on DAF-16-related transcriptional regulation are generally recognized as important in stress response and longevity (Heidler et al., 2010). As expected, *daf-16* (*p* < 0.001) and its downstream targets *sod-3* (*p* < 0.05) and *hsp-16.2* (*p* < 0.001) were all up-regulated with arbutin treatment relative to the control. Therefore, the results demonstrated that DAF-16 was vital to arbutin’s positive effect on lifespan and stress resistance in *C. elegans* (Figs. 6A–6C).

### Arbutin does not affect brood size in *C. elegans*

**Figure 7:**
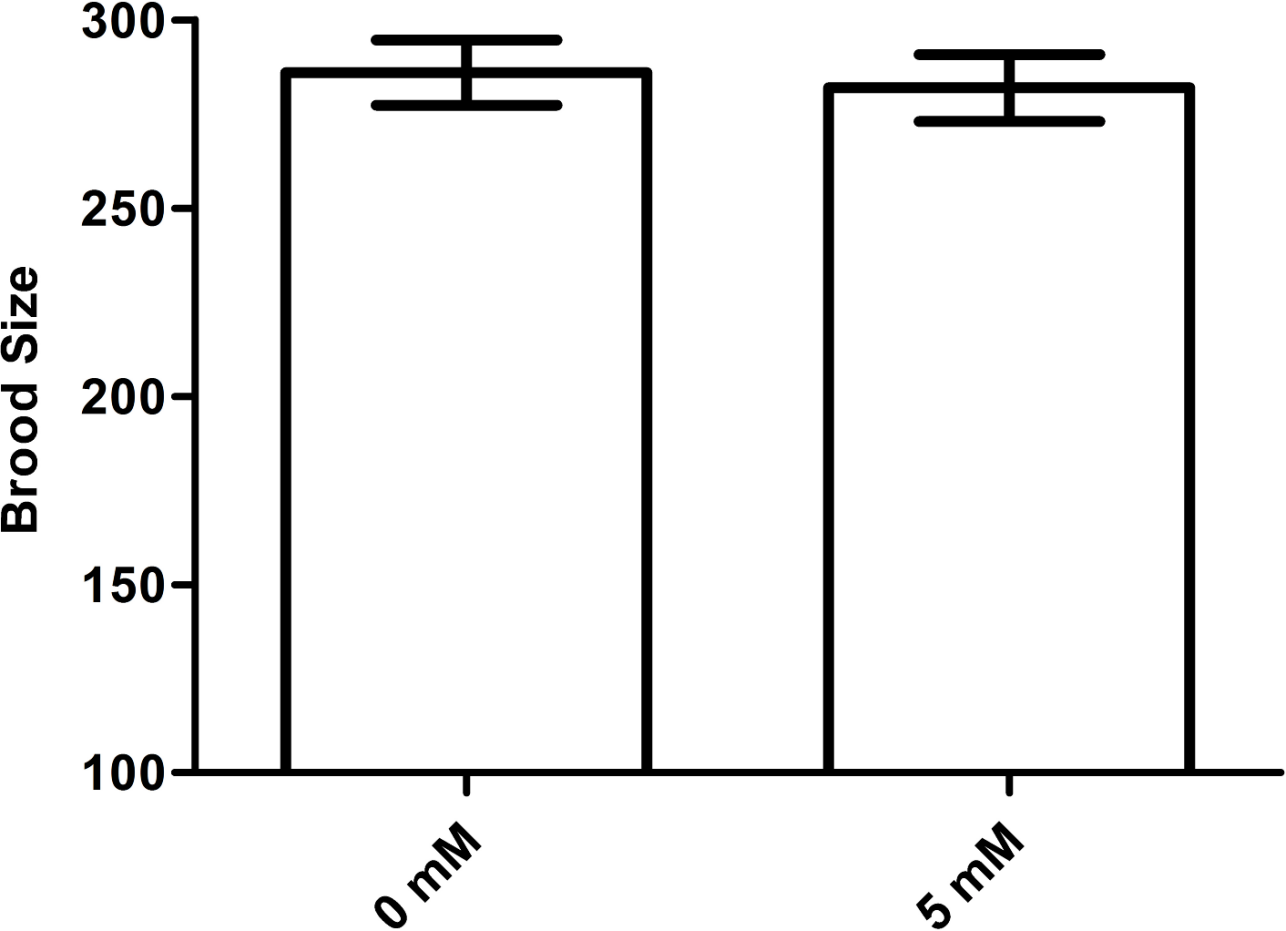
Effect of arbutin on brood size in *C. elegans*. There was no significant difference in the number of total progeny between arbutin-treated and untreated animals. *Error bars* represented the standard error of the mean (SEM) (*N* = 10 animals per group, *p* = 0.74).

An increase in lifespan is often correlated with a decrease in fecundity (Wang et al., 2017). To test whether arbutin adversely affected fecundity, we measured brood size for ten N2 animals in each group treated with either 0 mM (control) or 5 mM arbutin. The results showed that there was no significant difference between treatment group and control (Fig. 7, *p* > 0.05).

## Discussion

Arbutin (p-hydroxyphenyl- β-D-glucopyranoside) is a well-known tyrosinase inhibitor which has been widely used as a cosmetic whitening agent. It also plays an important role in free radicals scavenging and acts as a diuretic, as well as an anti-bacterial, anti-phlogistic, anti-tussive and anticancer agent (Mustapha et al., 2016). In this work, nematodes served as a model to test our hypothesis that arbutin could play a positive role in health and survival. To explore effects of arbutin, we dissolved it in deionized water and added it to worms’ food with several concentrations. Our work demonstrated that arbutin with suitable concentrations could extend the lifespan of *C. elegans* in a dose-dependent manner and the effect was sensitive to various stressors. Meanwhile, arbutin was observed to decrease ROS levels. Besides, *daf-16* played a role in the effects of arbutin on worms. Arbutin had no influenced on stress resistance in *daf-16* mutant. It not only increased DAF-16::GFP nuclear localization but also enhanced mRNA expression of *daf-16* and its downstream targets *sod-3* and *hsp-16.2* relative to controls. Therefore, the final results demonstrated that arbutin could improve lifespan and health of *C. elegans* which was related to *daf-16*.

Although arbutin was an anti-bacterial agent in *E. coil* OP50, worms were all fed with excess food, thus arbutin could not cause food deprivation. When treated with 0.5 mM, 2.5 mM and 5 mM arbutin, animals exhibited longer lifespan than control animals which were fed with *E. coil* OP50 alone in a dose-dependent manner. Conversely, worms treated with 10 mM and 20 mM arbutin died earlier than controls, indicating that 10 mM and 20 mM arbutin exhibited toxic effects on worms. Thus, within a specific range of suitable concentrations, arbutin could extend the lifespan of *C. elegans*. By comparison, we chose 5 mM arbutin for subsequent experiments. At the same time, 5 mM arbutin had no change of brood size of N2 worms, indicating that 5 mM also did no harm to animals’ reproduction and development.

To further confirm the influence of arbutin on lifespan, we treated worms with or without 5 mM arbutin, which had previously been revealed to be the most effective concentration for prolonging *C. elegans*’ lifespan. Worms were next exposed to a variety of stress circumstances. Regardless of the mode of stress applied, arbutin-treated animals exhibited greater resistance to stressors, including heat shock, juglone and UV-irradiation. These results indicated that arbutin could extend worms’ lifespan in natural environment and enhance resistance to stress.

After the effects of arbutin on *C. elegans* phenotypes were determined, we investigated arbutin’s mechanism of action. Previous research had established that oxidative damage was correlated with functional and metabolic decline during aging (Sena & Chandel, 2012). Moreover, while physiological ROS levels alter a cell’s redox state and play a role in mediation of cell signaling, pathological ROS levels can operate in concert with intracellular oxidative damage and activate several cell death pathways (Dai et al., 2014). Therefore, we measured ROS levels in worms pretreated with or without 5 mM arbutin and demonstrated that arbutin could reduce ROS levels effectively.

DAF-16, the orthologue of FOXO in *C. elegans*, is also a crucial transcription factor in stress resistance. We further investigated lifespan of *daf-16* mutant CF1038 under a variety of stressors. We found that the effect of arbutin on stress resistance of N2 worms disappeared in CF1038 worms under all condition of stressors. Meanwhile, we employed transgenic strain TJ356 (DAF-16::GFP) to test DAF-16 expression level in *C. elegans* and exhibited that arbutin could increase DAF-16 nuclear localization. Since moderate oxidative stress was reported to promote the expression of DAF-16 and its downstream target genes (Chiang et al., 2012), including *sod-3* and *hsp-16.2*, we designed primers for *daf-16*, *sod-3* and *hsp-16.2* and measured their relative mRNA levels. Subsequently, arbutin raised relative mRNA levels of *daf-16*, as well as its downstream transcription factors, as expected.

Thus, we concluded that suitable concentrations of arbutin could benefit lifespan extension and stress resistance in *C. elegans* through its antioxidant effects.

## Conclusions

In conclusion, our experiments demonstrated that arbutin significantly extends lifespan and improves stress resistance of nematodes. More importantly, its antioxidant activity might be related to DAF-16/FOXO-dependent pathways. Studies are underway to further explore the mechanisms by which arbutin may act on anti-aging and other health benefits.

##  Supplemental Information

10.7717/peerj.4170/supp-1 Data S1 Effect of arbutin on the lifespan of *C. elegans* Wild type animals (*N* = 120–182 in each group) were treated without (0 mM) or with low (0.5, 2.5 mM), moderate (5 mM, *p* < 0.001) and high (10, 20 mM) doses of arbutin at 20 °C from birth, when survival was monitored. The experiment was repeated multiple times and a representative trial is shown.

10.7717/peerj.4170/supp-2 Data S2 Effect of pretreatment with arbutin on resistance to stress in *C. elegans* Animals were treated with 5 mM arbutin from birth to 120 h at 20 °C and exposed to a variety of stressors. Worms pretreated with arbutin survived significantly longer after (A–B) 35 °C heat shock (*N* = 66–84 animals, *p* < 0.05), (C–D) exposure to 200 µM juglone (*N* = 90 animals, *p* < 0.001) or (E–F) UV irradiation at 1,000 J/m^2^ (*N* = 68–96 animals, *p* < 0.001). Each experiment is representative of three independent trials.

10.7717/peerj.4170/supp-3 Data S3 Effect of arbutin on ROS accumulation on *C. elegans* Wild type N2 animals treated with 5 mM arbutin accumulated less ROS than wild type N2 control animals (*N* = 24–25 times and 20 animals per group *p* < 0.001).

10.7717/peerj.4170/supp-4 Data S4 Effect of arbutin on resistance to stress in transgenic strain CF1038 *C. elegans* Animals were treated with 5 mM arbutin from birth to 120 h at 20 °C and exposed to a variety of stressors. There was no survival significance with worms pretreated with or without arbutin after (A–B) 35 °C heat shock (*N* = 74 animals, *p* > 0.05), (C–D) exposure to 200 µM juglone (*N* = 96 animals, *p* > 0.05) or (E–F) UV irradiation at 1,000 J/m^2^ (*N* = 85–88 animals, *p* > 0.05). Each experiment is representative of three independent trials.

10.7717/peerj.4170/supp-5 Data S5 Effect of arbutin on DAF-16::GFP nuclear localization on transgenic strain TJ356 Worms treated with 5 mM arbutin (A–B) displayed a fluorescence intensity that was significantly up-regulated (E) relative to the control group (C–D) (*N* = 10 animals per group, *p* < 0.001).

10.7717/peerj.4170/supp-6 Data S6 Effect of arbutin on relative mRNA levels of *daf-16*, *sod-3* and *hsp-16.2* of *C. elegans* *daf-16* (*P* < 0.001) and its downstream targets *sod-3* (*P* < 0.05) and *hsp-16.2* (*P* < 0.001) were all up-regulated with arbutin treatment relative to the control (*N* = 3 and 200 animals per group, *p* < 0.05).

10.7717/peerj.4170/supp-7 Data S7 Effect of arbutin on brood size of *C. elegans* There was no significant difference in the number of total progeny between arbutin-treated and untreated animals (*N* = 10 animals per group, *p* > 0.05).

## Additional Information and Declarations

### Data Availability

The following information was supplied regarding data availability: The raw data has been provided in the Supplemental Files.

## References

[ref-1] Abbas S, Wink M (2010). Epigallocatechin gallate inhibits beta amyloid oligomerization in Caenorhabditis elegans and affects the daf-2/insulin-like signaling pathway. Phytomedicine.

[ref-2] Balandrin MF, Klocke JA, Wurtele ES, Bollinger WH (1985). Natural plant chemicals: sources of industrial and medicinal materials. Science.

[ref-3] Chiang WC, Tishkoff DX, Yang B, Wilson-Grady J, Yu X, Mazer T, Eckersdorff M, Gygi SP, Lombard DB, Hsu AL (2012). C. elegans SIRT6/7 homolog SIR-2.4 promotes DAF-16 relocalization and function during stress. PLOS Genetics.

[ref-4] Dai DF, Chiao YA, Marcinek DJ, Szeto HH, Rabinovitch PS (2014). Mitochondrial oxidative stress in aging and healthspan. Longevity & Healthspan.

[ref-5] Harman D (1956). Aging: a theory based on free radical and radiation chemistry. Journals of Gerontology Series A: Biological Sciences and Medical Sciences.

[ref-6] Heidler T, Hartwig K, Daniel H, Wenzel U (2010). Caenorhabditis elegans lifespan extension caused by treatment with an orally active ROS-generator is dependent on DAF-16 and SIR-2.1. Biogerontology.

[ref-7] Henderson ST, Johnson TE (2001). *daf-16* integrates developmental and environmental inputs to mediate aging in the nematode Caenorhabditis elegans. Current Biology.

[ref-8] Hsu AL, Murphy CT, Kenyon C (2003). Regulation of aging and age-related disease by DAF-16 and heat-shock factor. Science.

[ref-9] Kaletta T, Hengartner MO (2006). Finding function in novel targets: C. elegans as a model organism. Nature Reviews Drug Discovery.

[ref-10] Lee RY, Hench J, Ruvkun G (2001). Regulation of *C. elegans* DAF-16 and its human ortholog FKHRL1 by the daf-2 insulin-like signaling pathway. Current Biology.

[ref-11] Li J, Ebata A, Dong Y, Rizki G, Iwata T, Lee SS (2008). Caenorhabditis elegans HCF-1 functions in longevity maintenance as a DAF-16 regulator. PLOS Biology.

[ref-12] Link P, Wetterauer B, Fu Y, Wink M (2015). Extracts of Glycyrrhiza uralensis and isoliquiritigenin counteract amyloid-beta toxicity in Caenorhabditis elegans. Planta Medica.

[ref-13] Livak KJ, Schmittgen TD (2001). Analysis of relative gene expression data using real-time quantitative PCR and the 2(-Delta Delta C(T)) Method. Methods.

[ref-14] Murphy CT, McCarroll SA, Bargmann CI, Fraser A, Kamath RS, Ahringer J, Li H, Kenyon C (2003). Genes that act downstream of DAF-16 to influence the lifespan of Caenorhabditis elegans. Nature.

[ref-15] Mustapha N, Mokdad-Bzeouich I, Maatouk M, Ghedira K, Hennebelle T, Chekir-Ghedira L (2016). Antitumoral, antioxidant, and antimelanogenesis potencies of Hawthorn, a potential natural agent in the treatment of melanoma. Melanoma Research.

[ref-16] Scalbert A, Manach C, Morand C, Remesy C, Jimenez L (2005). Dietary polyphenols and the prevention of diseases. Critical Reviews in Food Science and Nutrition.

[ref-17] Schlotterer A, Kukudov G, Bozorgmehr F, Hutter H, Du X, Oikonomou D, Ibrahim Y, Pfisterer F, Rabbani N, Thornalley P, Sayed A, Fleming T, Humpert P, Schwenger V, Zeier M, Hamann A, Stern D, Brownlee M, Bierhaus A, Nawroth P, Morcos M (2009). C. elegans as model for the study of high glucose- mediated life span reduction. Diabetes.

[ref-18] Sena LA, Chandel NS (2012). Physiological roles of mitochondrial reactive oxygen species. Molecular Cell.

[ref-19] Seo DH, Jung JH, Lee JE, Jeon EJ, Kim W, Park CS (2012). Biotechnological production of arbutins (alpha- and beta-arbutins), skin-lightening agents, and their derivatives. Applied Microbiology and Biotechnology.

[ref-20] Spencer JP, Abd El Mohsen MM, Minihane AM, Mathers JC (2008). Biomarkers of the intake of dietary polyphenols: strengths, limitations and application in nutrition research. British Journal of Nutrition.

[ref-21] Wang D, Hou L, Nakamura S, Su M, Li F, Chen W, Yan Y, Green CD, Chen D, Zhang H, Antebi A, Han JJ (2017). LIN-28 balances longevity and germline stem cell number in Caenorhabditis elegans through let-7/AKT/DAF-16 axis. Aging Cell.

[ref-22] Waterston RH, Brenner S (1978). A suppressor mutation in the nematode acting on specific alleles of many genes. Nature.

